# Hemipteran Pests of Sugarcane in North America

**DOI:** 10.3390/insects10040107

**Published:** 2019-04-14

**Authors:** Blake E. Wilson

**Affiliations:** Sugarcane Research Station, Louisiana State University Agricultural Center, 5755 LSU Ag Rd. St. Gabriel, LA 70776, USA; bwilson@agcenter.lsu.edu

**Keywords:** *Melanaphis sacchari*, *Sipha flava*, *Saccharosydne saccharivora*, *Perkinsiella saccharicida*, *Leptodictya tabida*, *Saccharum* spp.

## Abstract

Piercing-sucking herbivores (Insecta: Hemiptera) represent one of the greatest threats to agricultural production worldwide. Hemipteran pests directly injure plants as well as vector disease-causing plant pathogens. Production of sugarcane (*Saccharum* spp.) in North America is impacted by a complex of Hemiptera including the sugarcane aphid, *Melanaphis sacchari* Zehntner (Aphididae); yellow sugarcane aphid, *Sipha flava* (Forbes) (Aphididae); West Indian canefly, *Saccharosydne saccharivora* (Westwood) (Delphacidae); sugarcane delphacid, *Perkinsiella saccharicida* Kirkaldy (Delphacidae); and sugarcane lace bug, *Leptodictya tabida* (Herric-Schaeffer) (Tingidae). None of these pests is consistently damaging to large amounts of sugarcane acreage, but regional outbreaks are common. The biology, ecology, and pest management of these insects are discussed with emphasis on North America sugarcane production.

## 1. Introduction

Sugarcane is produced on more than 1.1 million ha in the U.S. and Mexico [1,2]. Sugarcane cultivation in the U.S. occurs in three distinct regions: Louisiana, Florida, and the Rio Grande Valley of Texas. Production of sugarcane in Mexico is concentrated on the coastal plains of both the Gulf and Pacific coasts with the states of Veracruz, San Luis Potosi, and Jalisco, accounting for the majority of sugar produced in the country [2]. The primary focus of entomological research in North America has focused on lepidopteran stem borers as this pest guild is considered the most economically important [3,4,5]. However, a complex of piercing-sucking insects (Hemiptera) that also attack sugarcane in this region has received relatively little attention from researchers. Much of the published literature on these pests is limited to isolated reports of outbreaks and new pest detections, while comprehensive sources of information are scarce. No works have examined the complex collectively or across geographical regions. The biology and ecology of five species of hemipteran pests attacking sugarcane in the U.S. and Mexico are reviewed herein.

## 2. The Sugarcane Aphid, *Melanaphis sacchari*

### 2.1. Morphology and Biology

**Figure 1:**
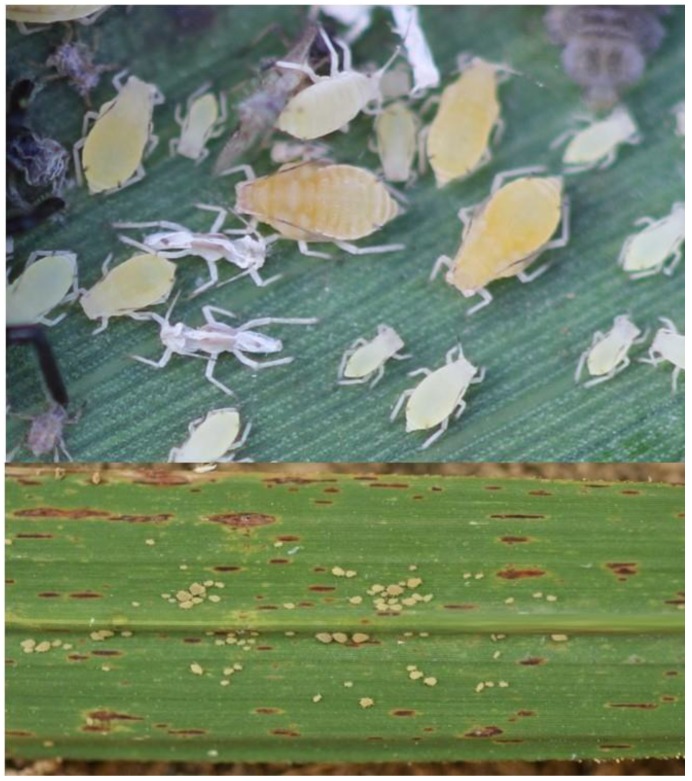
*Melanaphis sacchari* adults, nymphs, and exuvia (top); and colony on the underside of a sugarcane leaf (bottom). Photos ^©^ B.E. Wilson.

The sugarcane aphid, *Melanaphis sacchari* (Zehntner) (Hemiptera: Aphididae), was previously described as *Aphis sacchari* and *Longiunguis sacchari* before being placed in its current genus [6]. *Melanaphis sacchari* is a small aphid (<2 mm), generally beige in color, but displays significant color variation to include colonies with shades of pink, brown, and yellow aphids depending on host plants and environmental conditions (Figure 1) [7]. Dark-colored tarsi, cornicles, and antennae are features commonly used to distinguish *M. sacchari* from other aphid species infesting the Poaceae [7]. The species has siphunculi that are slightly longer than its cauda as well as terminal processes that are much longer than the last antennal segment [6].

The life cycle of *M. sacchari* is anholocyclic with colonies feeding on primary hosts during the spring and summer, and overwintering only in regions where host plants remain green through the winter, including Mexico, southern Texas, Florida, and the southern-most regions of Louisiana, Mississippi, and Alabama bordering the Gulf Coast [7]. Reproduction is characterized as parthenogenic viviparity. The North American population is primarily asexual, but sexual reproduction has been reported in Asia [8,9,10]. Development to adulthood is highly variable depending on temperature and host plants generally ranging from 4.3 to 12 days [6,11,12].

### 2.2. Distribution and Host Plants

*Melanaphis sacchari* has a near global distribution occurring in South Africa, India, South East Asia, China, Central America, South America, Australia, and most recently, North America. The species was first discovered in the U.S. in Florida sugarcane in 1922 [13]. It was reported in Louisiana sugarcane in 1999 [14]. While *M. sacchari* has received more attention globally as a pest of sorghum (*Sorghum bicolor*), the U.S. population remained largely confined to sugarcane until 2013 when widespread outbreaks occurred in sorghum throughout the Mid-South region [7]. Population genetic studies suggest this host switch resulted from the introduction of a new lineage that is genetically distinct from specimens collected from Louisiana sugarcane in 2007 [15]. In addition to sugarcane and *Sorghum* spp., *M. sacchari* can develop on grasses (Poaceae) in the genera *Oryza*, *Panicum*, *Pennisetum*, and *Echnicholoa* [6].

### 2.3. Ecology, Pest Status, and Management in Sugarcane

Feeding on sugarcane leaves by *M. sacchari* causes minimal symptomology until heavy infestations lead to growth of black sooty mold on leaves covered in aphid-produced honeydew. Devastating impacts of *M. sacchari* infestations on yields of sorghum are common [8], but direct effects of feeding on sugarcane yields are not well understood. Aphid feeding reduces chlorophyll content in leaves and removes amino acids [11]. Formation of sooty mold results from growth of a complex of fungi on honeydew, and is thought to reduce the surface area available for photosynthesis. Presumably, the development of sooty mold reduces photosynthesis negatively impacting plant growth. However, it is not clear how these factors affect sugar yields, and yield losses in North American sugarcane have not been documented. The aphids’ vector potential is of greater importance to sugarcane producers. Viruses which are vectored by *M. sacchari* include persistent sugarcane yellow leaf virus (ScYLV), red-millet leaf virus, and sugarcane mosaic virus [6,16]. Its ability to efficiently transmit ScYLV [16] is considered the greatest threat from this pest as the virus has caused yield losses up to 14% in Louisiana sugarcane [17]. Incidence of ScYLV in Louisiana is spatial and temporally associated with infestations of *M. sacchari*, suggesting that this species is the primary vector of the disease [18]. However, generally low incidence of the disease even with high prevalence of vectors suggests the inoculum pressure is low in Louisiana [18]. The importance of ScYLV in Louisiana has declined in recent years since the virus was added to certification standards for micropropagated seedcane [18]. ScYLV remains prevalent in Florida sugarcane with approximately 89% of fields infected [19] and is responsible for an estimated yield loss of 4–7% throughout the industry [20]. Another economically important virus transmitted by *M. sacchari* is the sugarcane mosaic virus [16], which has potential to dramatically decrease yields of susceptible varieties. Although *M. sacchari* is a competent vector of sugarcane mosaic virus, other aphid species which are more transient in sugarcane fields are primarily responsible for virus spread [21]. Management of aphid vectors is not effective at reducing virus incidence and disease-resistant cultivars are the primary management strategy for these diseases [21].

Populations of *M. sacchari* frequently reach very high densities in susceptible sugarcane cultivars in Louisiana, though infestations are typically localized and not widespread. Populations typically peak in late July with infestations of over 500 aphids per leaf having been recorded, but high populations do not persist [22,23,24]. Research into management of *M. sacchari* has identified effective chemical controls, resistant cultivars, and biological control agents. Effective insecticides which have been identified include neonicotinoids and related compounds such as sulfoxaflor and flupyradifurone [23,24,25], but these products are not registered for use in U.S. sugarcane. Only pyrethroids are labeled for *M. sacchari* control, and these products have been shown to be ineffective. In some cases, pyrethroid applications have led to pest resurgence presumably from reductions of natural enemies [26,27]. Insecticidal control of *M. sacchari* is not recommended due to unavailability of effective products, tendency for populations to decline naturally, and lack of evidence of benefits to yields [28].

Because of the limited chemical control options, cultivar resistance has been the focus of recent *M. sacchari* management research in sugarcane. Resistant cultivars including HoCP 91-555 can suppress *M. sacchari* population growth [11] and show reduced infestations in the field [29]. This resistance is thought to be based on reduced concentrations of free amino acids which are essential to the *M. sacchari* growth and reproduction [30]. While other mechanisms may also influence this resistance, previous studies did not find evidence that antixenosis or tolerance are involved [11]. Future research should examine levels of resistance to *M. sacchari* among commercial cultivars which are currently produced on substantial acreage in the U.S.

Numerous indigenous and exotic natural enemies are known to feed on *M. sacchari* in North America. Many of those which have been identified from sorghum fields in Texas [31] are likely also present in sugarcane fields. Syrphid and coccinellid larvae were the greatest contributors to mortality with chrysopids and aphelinids contributing to a lesser extent, although these interactions varied among agro-ecosystems and may not apply to sugarcane [31]. The coccinellid, *Diomus terminatus* Say is known to be present in Louisiana sugarcane fields, where it is an important natural enemy of aphids including *M. sacchari*. This predator was shown to consume 30 *M. sacchari* nymphs during larval development with adult beetles consuming approximately 19 nymphs daily [32]. This species can be reared on aphids in the laboratory and may have potential for use as an augmentative biological control agent for aphids in sugarcane [32,33]. Entomopathogenic fungi recorded infecting *M. sacchari* in sorghum [34] may also contribute to population declines in sugarcane, especially under conditions of high aphid population densities.

Extensive research efforts into *M. sacchari* management in sorghum after the 2013 outbreak will potentially yield novel management strategies which could be applied to sugarcane as well. Future research efforts should examine potential for new effective chemical controls and continued assessment of cultivar resistance. Studies are urgently needed to determine the relationship between aphid densities and sugar yields.

## 3. Yellow Sugarcane Aphid, *Sipha flava*

### 3.1. Morphology and Biology

**Figure 2:**
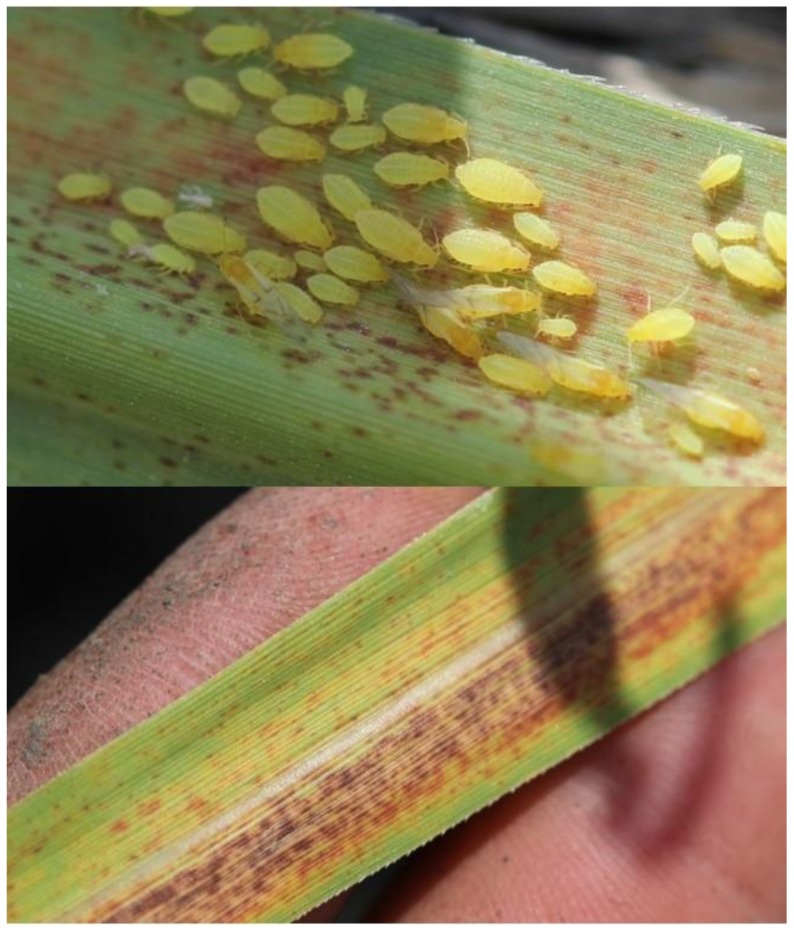
Yellow sugarcane aphid, *Sipha flava*, nymphs, adults, and alates (top); and reddening/yellowing of sugarcane leaves caused by *S. flava* feeding (bottom). Photos: ^©^ B.E. Wilson.

Yellow sugarcane aphids, *Sipha flava* (Forbes) (Figure 2 (top)), are small (<2 mm), brightly colored aphids with numerous hairs covering the head, thorax, and abdomen. Rows of dark spots dot the abdomen and the cornicles are reduced in size relative to other aphid species. Alate forms maintain bright yellow abdomens, but the head and thorax are darker in color [35]. The species is parthenogenic throughout the year in warmer regions of its range, but sexual forms occur where low winter temperatures induce oviparity [8]. Development to adulthood takes 8–15 days and is highly dependent on temperature and host plants. Development occurs more rapidly on sorghum than on sugarcane [11,36].

### 3.2. Distribution and Host Plants

*Sipha flava* is widely distributed in the New World and is known to occur throughout the contiguous U.S. as far north as New York and Washington State as well as in Mexico, Central and South America, and the Caribbean [8]. The species has more recently become established as an invasive pest in parts of Africa [37]. The species is known to infest sugarcane in all regions of its range where the crop is cultivated [8]. *S. flava* also utilizes a number of crop and non-crop grasses. In addition to sugarcane, crop hosts that may be attacked include sorghum, rice (*Oryzae sativa*), and wheat (*Triticum aestivum*). Non-crop members of the same genera as well as plants in the genera *Digitaria, Hordeum, Panicum, Paspalum,* and *Pennisetium* are also attacked [8].

### 3.3. Ecology, Pest Status, and Management in Sugarcane

*Sipha flava* most commonly attacks young sugarcane prior to the development of multiple internodes [35,38]. The aphids feed on the underside of immature leaves causing yellowing/reddening of tissues (Figure 2 (bottom)) leading to premature senescence or chlorosis. Feeding on young plants can cause major damage under high levels of infestation. Chlorosis of 2–3 leaves early in the growing season has been reported to reduce sugar yields up to 6% with losses of up to 19% occurring when >6 leaves are chlorotic [39]. Other studies have shown substantially reduced plant height and tillering resulting from *S. flava* feeding [40]. Thus, yield losses are likely attributable to reduced cane tonnage from reduced growth early in the growing season. In addition to direct yield losses from aphid feeding, *S. flava* is also a competent vector of sugarcane mosaic virus [9].

In most sugarcane producing regions, *S. flava* is a sporadic pest and generally not of great economic importance [35,38,41]. However, favorable climatic conditions can result in outbreaks occur. Dry weather has been associated with *S. flava* outbreaks in sugarcane in all parts of the pest’s range [35,38], and heavy rainfall is thought to mechanically control infestations by washing aphids on to the ground where they drown or become immobilized in mud [41]. High humidity and rainfall are also conducive to infection by entomopathogenic fungi [42]. Isolated outbreaks are also thought to be associated with high densities of weedy grass hosts in close proximity to sugarcane fields [41]. Infestations in Louisiana are common from April to June, but generally decline naturally before they reach damaging levels [38]. Infestations in Florida occur on newly planted sugarcane in the fall and on new growth occurring in the spring [35]. Cultivation of susceptible cultivars in Florida led to instances of severe infestations, but the pest has since declined in economic importance [39,40].

Management of *S. flava* is achieved with resistant cultivars, chemical controls, and biological control. Growth and development of *S. flava* on resistant sugarcane cultivars is reduced by 1.5–3-fold relative to susceptible cultivars, though mechanisms of resistance have not been studied. Feeding by *S. flava* on resistant cultivars also caused less chlorophyll loss than susceptible cultivars [11]. Chemical controls are not consistently recommended as there is little evidence that insecticide applications targeting *S. flava* will improve yields or reduce virus transmission. Further, there is concern that insecticides may disrupt natural enemy populations resulting in pest resurgence or secondary pest outbreaks [38]. Products which can effectively control this pest have been identified including imidacloprid, sulfoxaflor, and flupyridifuron [24]; however, only pyrethroids are labeled for *S. flava* control in sugarcane in the United States. Many of the same biological control agents that attack *M. sacchari* also feed on *S. flava* [40]; however, these interactions have not been well studied.

## 4. The West Indian Canefly, *Saccharosydne saccharivora*

### 4.1. Morphology and Biology

**Figure 3:**
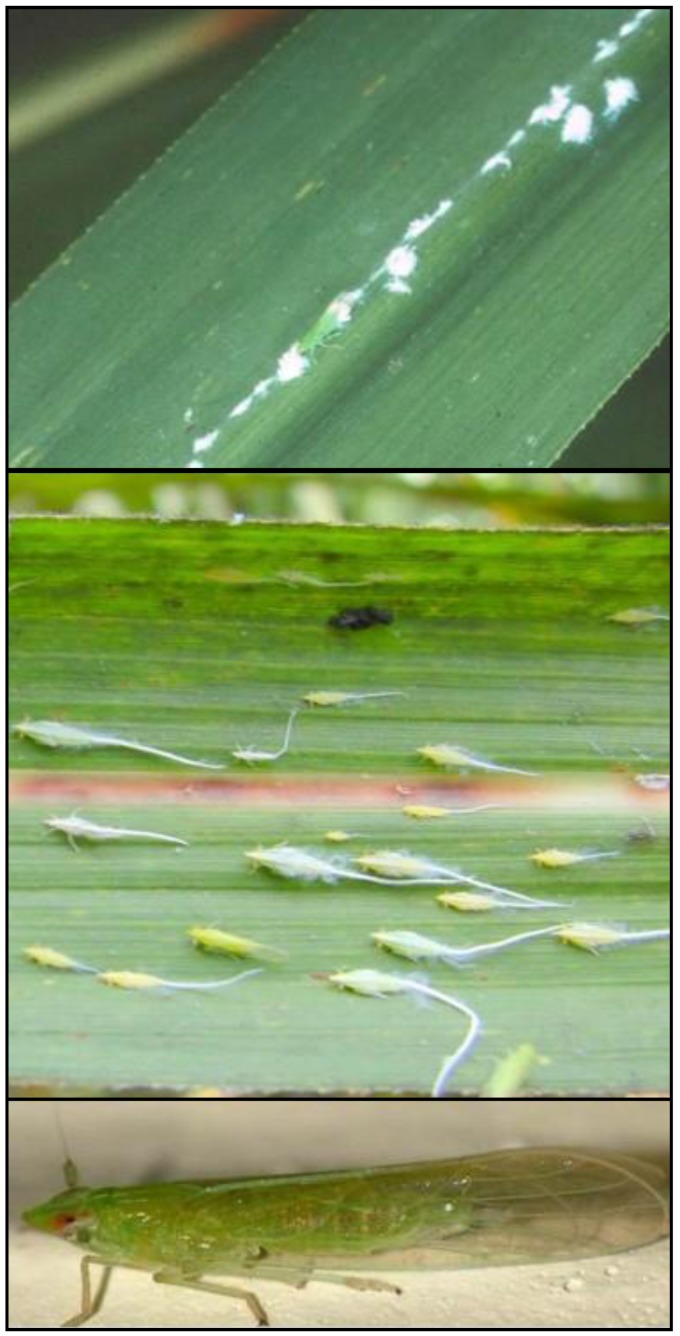
*Saccharosydne saccharivora* eggs (top), nymphs (middle), and adult (bottom). Photos: ^©^ B.E. Wilson.

Eggs of the West Indian canefly, *Saccharosydne saccharivora* Westwood (Hemiptera: Delphacidae), are laid within tissues of the abaxial side of leaves and then covered in protective webbing (Figure 3 (top)). The eggs appear as cottony material typically occurring on the mid-ribs of sugarcane leaves and can easily be observed in the field [43,44]. However, because the protective webbing remains present after eclosion, it is not easy to distinguish between newer and older oviposition events. The egg stage lasts from 13 to 23 days with peak hatch occurring at 15–16 days [44]. After eclosion from eggs, small yellow nymphs with a characteristic “tail” begin feeding on sugarcane leaves (Figure 3 (center)). The nymphs are highly mobile often scurrying or jumping off leaves if disturbed. The nymphs progress through five nymphal stadia resembling adults more with each subsequent molt. Development from nymph to adult averages 17.6 and 19.2 days for males and females, respectively. Developmental duration is dependent on host quality [44]. Adults are bright green delphacids approximately 3–5 mm in length with clear wings and red eyes (Figure 3 (bottom)) [44]. Adult females and males are easily distinguished by size (males are smaller), external genitalia, or the presence of cottony wax posterior on females. The sex ratio is approximately equal. Adult longevity is approximately 1 week for males and up to 4 week for females [44]. Like the nymphs, adults are highly mobile and will fly off leaves when disturbed. Overlapping generations result in occurrence of eggs, nymphs, and adults on the same leaves [43]. Adults are present at lesser densities than nymphs and are thought to emigrate out of infested fields when population densities are high [45,46,47].

### 4.2. Distribution and Host Plants

*Saccharosydne saccharivora* is widely distributed throughout the Americas and the Caribbean wherever sugarcane is grown. It has historically been a major pest of sugarcane in Jamaica [44,45,46]. The pest has been documented infesting sugarcane in the U.S. in Florida, Louisiana, and Texas [47,48,49] as well as the islands of Jamaica, Cuba, Puerto Rico, and other regions [44]. Reports from Latin America include Mexico, Ecuador, Honduras, and others [45]. It is not clear if any of these populations resulted from an introduction or if these areas constitute its native range. While in many areas it was not reported until recent decades, the sporadic nature of outbreaks in much of its range would possibly allow the pest to persist unnoticed for many years. Similarly, the insect could be present in low numbers on non-crop hosts in areas outside of its current known range such as southern Mississippi and Alabama.

Sugarcane seems to be the most suitable host of *S. saccharivora*, as populations on non-crop hosts have not been observed to reach comparable densities. Populations of adults and nymphs have been observed on *S. halepense*, in the U.S. [47] and Caribbean suggesting this host plant can support completion of the life cycle [44]. Metcalfe also identified *Panicum virgatum* and two species of *Andropogon*, as non-crop hosts capable of supporting development to adulthood [44]. Numerous other grasses have been identified to support feeding by adults, but these are thought to be only temporary hosts that will not support nymphal development [44]. It is not clear what role these hosts have in the ecology of *S. saccharivora* and more research into its host range and seasonal utilization is needed. It has not been reported to attack economically important grasses despite sorghum, corn, rice, and others frequently grown in proximity to infested sugarcane fields [43], suggesting that its pest potential is limited to sugarcane.

### 4.3. Ecology, Pest Status, and Management in North American Sugarcane

The ecology of *S. saccharivora* has been well studied in the Caribbean [44,45,46], and to a lesser extent the U.S. [47,49]. The pest has a demonstrated preference for young sugarcane in both regions; however, infestations have been observed to persist to maturing cane in late summer in the U.S. when younger cane is not abundant [47]. Population dynamics throughout its range are sporadic and outbreaks of immense populations have frequently been documented. The factors that influence these outbreaks are not clear, although environmental conditions such as temperature and precipitation are likely key parameters. Widespread outbreaks have been reported from Louisiana in 1945 [50], 1969 [51], and 1997 [52] in addition to more frequent outbreaks occurring there in 2012, 2016, and 2017 [47]. It has been suggested that because of the prevalence of *S. saccharivora* in tropical regions, its populations are likely limited in temperate regions of the U.S. by cold winter temperatures. Indeed, many of the Louisiana outbreaks followed warm winters, but the species is typically present in low numbers in most fields in Louisiana every year [43]. Presumably, this is also the case in other areas of its range where previous outbreaks have occurred.

**Figure 4:**
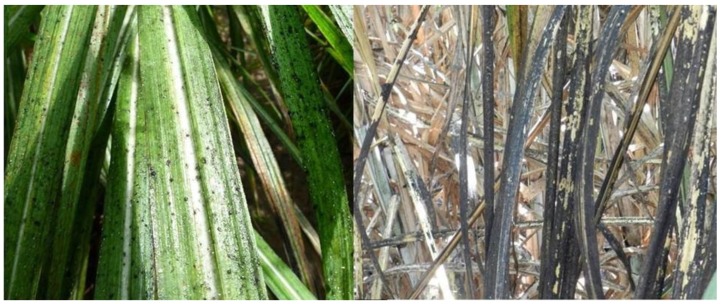
Sugarcane leaves covered in honeydew produced by *S. saccharivora* (left), and resulting sooty mold (right). Photos: ^©^ B.E. Wilson.

Infestations generally occur first in young sugarcane and frequently persist for many weeks. Under outbreak conditions, pest densities continue to rise through the spring and early summer. Peak densities of >100 nymphs and adults per leaf have been observed during June and July in Louisiana [47]. Populations recorded in Texas peak in May at much lower levels than those reported in Louisiana [49]. Similarly, populations in Florida persist at low levels consistently, but widespread outbreaks like those observed in Louisiana and the Caribbean have not been observed [48]. As with the aphid species, when *S. saccharivora* feeds on sugarcane sap, excess sugars are secreted as honeydew. Accumulation of large amounts of honeydew as infestations persist leads to the development of extensive sooty mold in the lower canopy (Figure 4) [45,46,47]. Heavy rain events can often reduce infestation levels, and have the added benefit of washing off honeydew before extensive sooty mold develops [43].

Infestations during outbreaks often cause such extensive growth of sooty mold, that considerable reductions in plant growth are thought to be unavoidable. However, evidence of yield losses resulting from *S. saccharivora* is scant and frequently inconclusive. Extensive documentation of high *S. saccharivora* populations in the Caribbean has led to it being frequently referred to as an important economic pest [44,45,46]. Unfortunately, none of these studies provided data-based evidence that infestations result in observable yield impacts. Attempts to document yield impacts during the 2012 and 2016 Louisiana outbreaks produced inconsistent results. In the study by Wilson et al. [47], a 36% loss in sugar yields was observed in non-treated plots relative to insecticide-protected plots in a single experiment. Yield was impacted to a lesser extent in large plot trials, but *S. saccharivora* infestations were not quantified in that trial. No impacts were observed in four additional experiments reported therein which had comparable levels of infestation. Furthermore, the Louisiana sugarcane crop yield in 2012 was among the highest ever recorded despite widespread *S. saccharivora* infestations going largely uncontrolled [47]. Thus, it was concluded that *S. saccharivora* infestations have potential to impact sugar yields, but that this highly variable relationship is likely dependent on numerous factors. Impacts of *S. saccharivora* on yields are thought to be influenced by infestation timing, duration, sugarcane variety, and environmental conditions. While determination of the pest status of *S. saccharivora* will require further research efforts, pest management research has provided evidence that effective controls can be implemented to respond to outbreaks.

Numerous chemical insecticides including λ-cyhalothrin, imidacloprid, acetamiprid, sulfoxaflor, flupyradifurone, and others have been shown to effectively reduce *S. saccharivora* infestations in sugarcane [52,53,54,55]. In nearly all trials to date, a single application provided effective control and no infestation recovery was observed. Products evaluated which did not provide control include buprofezin, tebufenozide, and chlorantraniliprole [47]. Further research into application timing and economic thresholds is needed to refine the use of chemical controls against *S. saccharivora* in sugarcane.

Sooty mold ratings demonstrating a range of susceptibility is present among Louisiana sugarcane cultivars [47]. These results are consistent with observations from Jamaica; however, factors such as growth stage and fertilization were reported to have a greater influence on *S. saccharivora* populations than sugarcane cultivar in those studies [45,46]. Examination of factors influencing this resistance should be investigated. Host plant preferences could influence oviposition sites. Alternatively, resistant cultivars could impede growth and development of nymphs as is frequently observed with other hemipteran pests.

Little research into biological control has been conducted. Metcalfe et al. [45,46] reported that populations of the *S. saccharivora* parasitoid, *Stenocranophilus quadratus* Pierce (Strepsiptera: Halictophagidae), frequently reached sufficient levels to cause rapid declines in pest infestations in Jamaica. Parasitism by a strepsipteran on *S. saccharivora* nymphs was frequently observed in Louisiana, but not at levels which would exert major influence on pest population dynamics [47,55]. Although males were not collected for species determination, observed parasitized nymphs were consistent in appearance to those described by Metcalfe [46]. Generalist predators including coccinellid beetles, spiders, and predatory hemipterans were frequently observed preying on *S. saccharivora* in Louisiana, but the influence of predation on population dynamics is unknown. Natural enemies observed attacking *S. saccharivora* in Florida include *S. quadratus*, *Tytthus parviceps* (Rueter) (Hemiptera: Miridae), *Psuedogonatopus variistriatus* (Hymenoptera: Dryinidae), and *Paracentrobia* spp. (Hymenoptera: Trichogrammatidae) [48].

Future research efforts into the pest status of *S. saccharivora* should focus on determination of factors which influence population dynamics leading to epidemic outbreaks as well as elucidation of pest density-yield loss relationships. Difficulties researching this pest in North America related to inconsistent field populations could be overcome through examination of reproductive potential and plant injury levels in a greenhouse setting. Indeed, greenhouse colonies of *S. saccharivora* were established and maintained during early research efforts in the Caribbean [44,45].

## 5. The Sugarcane Leafhopper *Perkinsiella saccharicida*

### 5.1. Morphology and Biology

**Figure 5:**
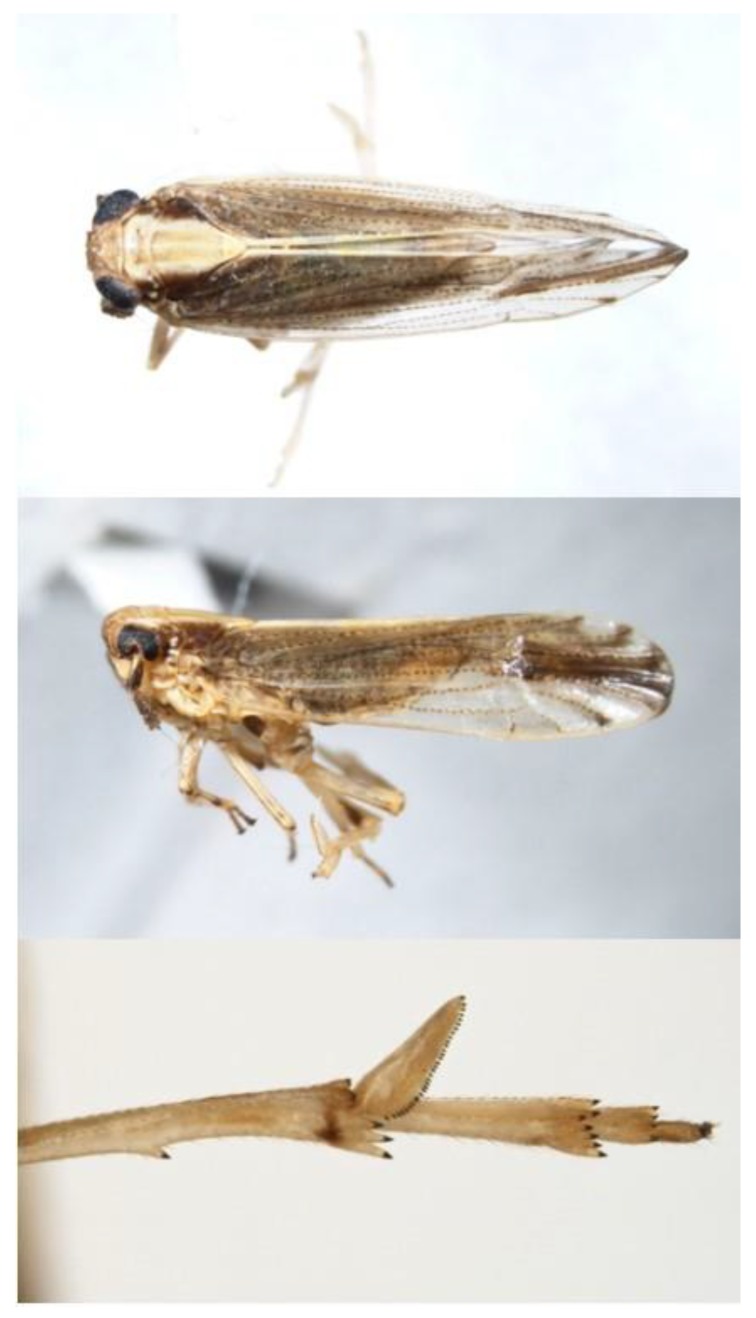
*Perkinsiella saccharicida* adult dorsal view (top), lateral view (middle), and hind tibia spur (bottom). Photos: ^©^ C. Bartlett and K. Shropshire, University of Delaware.

The sugarcane leafhopper, *Perkinsiella saccharicida* Kirkaldy (Hemiptera: Delphacidae), is a brown planthopper (4.5–6 mm) with light-colored dorsal markings on the head and thorax (Figure 5) [56]. The species can be identified by the presence of short clubbed antennae and a flattened spur on the apex of the hind tibia [56]. Substantial polymorphisms exists among adults with both macropterous and brachypterous individuals of both sexes having been observed [57]. There is no record, however, of brachypterous adults from North America. Nymphs resemble adults in structure and coloration, but lack wings.

Elongate eggs (1.0 × 0.3 mm) are laid in sugarcane leaf mid-ribs and covered with white waxy substance. Females lay up to 300 eggs in masses of 2–12 eggs over their roughly 30-day adult life span [58]. Development through five nymphal stages occurs over a period of 25–30 days [59]. The species is multivoltine in North America with multiple life stages present throughout the year [58].

### 5.2. Distribution and Host Plants

The native range of *P. saccharicida* is believed to be Papua New Guinea, and likely spread to other regions of Asia and Pacific islands following the introduction of sugarcane [60]. The pest has since been introduced to nearly all tropical and sub-tropical regions where sugarcane is produced including Asia, Africa, Australia, and the Americas [61]. It was first reported in the continental U.S. in Florida sugarcane in 1982 [62], and its range expanded to Texas and Mexico by 1991 [63] and Louisiana by 1994 [64].

The primary and most widely utilized host plant of *P. saccharicida* is sugarcane [60]. The species has also been recorded on sedges (*Carex* sp.), rice, and Arabica coffee (*Coffea arabica*) [58], although the degree to which these alternative hosts support nymphal development is unknown. These and other alternative hosts likely support populations of *P. saccharicida* as the pest has been observed to be attracted to lights in areas distant from sugarcane production including Georgia [65].

### 5.3. Ecology, Pest Status, and Management in North American Sugarcane

Much about the ecology of *P. saccharicida* in North American sugarcane is not well understood. The species is common in sugarcane fields in Louisiana [64], Florida [62], Texas, and Mexico [49,63], but it is generally not considered an economic pest in those regions. Densities greater than one per stalk have not been reported in Texas or Louisiana, but populations reached 37 nymphs and adults per stalk in Florida [62]. Populations in North America are greatest in maturing sugarcane in mid- to late-summer with infestations persisting into October and November [49,64]. Much higher densities of hundreds of nymphs per stalk have been reported from areas of the Pacific and Australia [66]. It is not clear what factors limit populations in North America relative to other regions, but climate and natural enemies are likely key factors. In Louisiana, high populations were recorded from fields in more southern, coastal regions with warmer temperature, suggesting that cold may be a limiting factor [64].

Natural enemies observed attacking *P. saccharicida* in Florida include *T. parviceps*, which also attacks *S. saccharivora*, as well as the *Anagrus* spp. (Hymenoptera: Mymaridae) parasitoids [48]. Two species of *Tytthus* are thought to provide some appreciable population suppression in Australia [66], but the role of these predators in controlling *P. saccharicida* in North America has not been examined.

Management strategies of *P. saccharicida* in North America have not been investigated because of the relatively minimal economic importance of the species. Globally, research has focused on its role as a vector of Fiji leaf gall virus [67], which has never been detected in North or South America [58]. Differences in preference and infestation levels among sugarcane cultivars have been documented in Texas [63] and Australia [66], but utilization of resistant cultivars as a management strategy has not been realized.

Pest management research for *P. saccharicida* is scarcely warranted in North America as damaging infestations have not been observed in any sugarcane fields during the approximately 30 years it has been established on the continent. Monitoring populations of *P. saccharicida* for potential to transmit Fiji leaf gall virus should be conducted periodically as introduction of this virus could have major impacts to sugarcane throughout the Americas.

## 6. Sugarcane Lace Bug, *Leptodictya tabida*

### 6.1. Morphology and Biology

**Figure 6:**
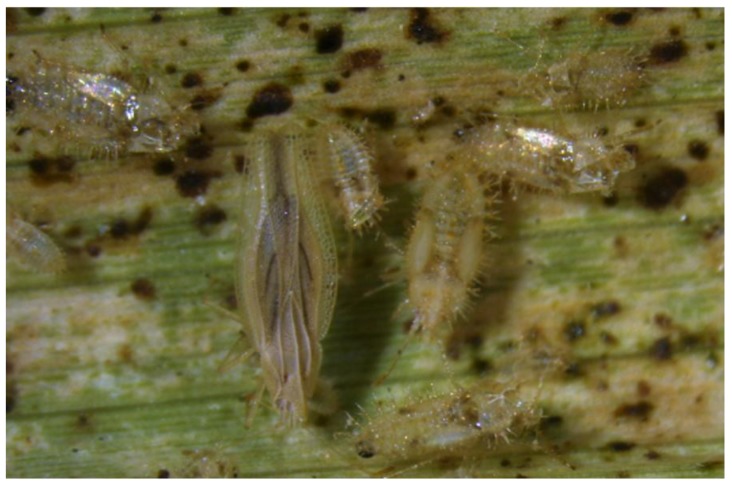
*Leptodictya tabida* adult and nymphs feeding on a sugarcane leaf. Photo: ^©^ D. Sekula, Texas A&M AgriLife.

The sugarcane lace bug, *Leptodictya tabida* (Herrich-Schaeffer) (Hemiptera: Tingidae) was originally described from Mexico as *Monanthia tabida*, and was subsequently redescribed by Stal and placed in the genus *Leptodictya* [68]. Adults are flat and oblong (3.5–4 mm in length) [69] with white or cream coloration (Figure 6). Five spines are apparent on the head. Antennae are long and thin. Elytra are transparent with the characteristic lace-like wing veination typical of tingids and extend beyond the abdomen. Nymphs are more yellowish in coloration with numerous white spinules protruding in all directions. [68,69].

Small dark-colored eggs are deposited in plant tissue on the underside of leaves, and are too small for easy observation in the field [69]. The egg stage lasts 16–20 days. Nymphs develop through five stadia maturing to adulthood in approximately 15 days [69,70]. Nymphal wing buds become visible in later stadia [68].

### 6.2. Distribution and Host Plants

*Leptodictya tabida* is widely distributed in sugarcane-producing regions in the Americas and the Caribbean. The species is thought to be native to Mexico and Central America as this region is where initial species descriptions were reported in the mid-19th century [68]. It was reported from Texas as early as 1925. It was also detected in Florida in 1990 [70]. The full extent of its distribution in the Americas has not been determined, but the species is not thought to occur in Louisiana. The species was introduced into Hawaii in the early 1980s [71].

While host range testing has not been conducted for *L. tabida*, the species likely has a broad host range including many species of Poaceae. It has been reported on corn, *Zea mays* L., *S. halepense*; barnyard grass, *Echinochloa crus-galli* (L.), Guinea grass, *Panicum maximum*, and other grasses [69]. 

### 6.3. Ecology, Pest Status, and Management in North American Sugarcane

Adults and nymphs of *L. tabida* feed in groups on the underside of sugarcane leaves, causing discoloration [72]. In Mexico, the species is regarded as a minor pest. It is reported by VanZwaluwenburg [5] to occur in high numbers on “older cane”, but it is not clear if this refers to crop maturity or older sugarcane stubble. The species was reported to be infesting sugarcane in south Texas in increasing levels in the early 2000s [73], but has not developed into an economic pest in which pest management tactics are employed [74]. The species has also been reported to infest sugarcane in high numbers in Florida where it can cause substantial discoloration of leaves and is presumed to be reducing photosynthesis. Although quantitative studies have not been conducted, high level infestations are reported to have yield-reducing potential [72]. In both Texas and Florida, differences in infestations levels among sugarcane varieties have been reported. Interestingly, no parasitoids or predators have been reported in its native (Texas) or introduced (Florida) range [5,72]. An attempt at classical biological control was conducted in Florida by importing *Erythmelus* sp. (Hymenoptera: Mymaridae), but establishment was unsuccessful [69].

No management tactics have been developed and the species is not considered a major pest of sugarcane at this time. If the species becomes a greater threat, future research should examine potential integrated pest management strategies. Establishment of *L. tabida* in Louisiana could have major impacts to the nearly $1 billion USD sugarcane industry there.

## 7. Conclusions

The five hemipteran species reported to infest North American sugarcane are considered the greatest threats to the crop’s production following lepidopteran stemborers and soil-dwelling Coleoptera due to their potential for sporadic outbreaks and isolated impacts. Numerous additional hemipteran insects have been reported to feed on sugarcane occasionally, but never reach concerning levels to warrant further investigation. A more complete list of these insects was published in the late 1980s [48], and additional species have likely been observed in more recent years. Despite widespread occurrence of these species in sugarcane production systems, none of these insects is considered a consistent threat to sugarcane in any region of North America. The inability to document substantial yield losses from sap-feeding insects in sugarcane is not uncommon. The most serious threats from hemipteran insects appear to be related to their roles as vectors of pathogens.
